# Randomized controlled double-blind trial of methylprednisolone versus placebo in patients with post-COVID-19 syndrome and cognitive deficits: study protocol of the post-corona-virus immune treatment (PoCoVIT) trial

**DOI:** 10.1186/s42466-024-00311-w

**Published:** 2024-03-21

**Authors:** Christiana Franke, Vanessa Raeder, Fabian Boesl, Benno Bremer, Lucas C. Adam, Ameli Gerhard, Irina Eckert, Anneke Quitschau, Anne Pohrt, Susen Burock, Lisa Bruckert, Carmen Scheibenbogen, Harald Prüß, Heinrich J. Audebert

**Affiliations:** 1 Department of Neurology and Experimental Neurology, Corporate Member of Freie Universität Berlin and Humboldt Universität zu Berlin, Charité – Universitätsmedizin Berlin, Charité Campus Benjamin Franklin, Hindenburgdamm 30, 12203 Berlin, Germany; 2 Corporate Member of Freie Universität Berlin and Humboldt-Universität zu Berlin, Institute of Biometry and Clinical Epidemiology, Charité - Universitätsmedizin Berlin, Charitéplatz 1, 10117 Berlin, Germany; 3 Corporate Member of Freie Universität Berlin and Humboldt Universität zu Berlin, Clinical Trial Office, Charité - Universitätsmedizin Berlin, Berlin, Germany; 4 Department of Neurology, Corporate Member of Freie Universität Berlin and Humboldt Universität zu Berlin, Charité - Universitätsmedizin Berlin, Charitéplatz 1, 10117 Berlin, Germany; 5 Deutsches Zentrum für Neurodegenerative Erkrankungen (DZNE), Berlin, Germany; 6 Institute of Medical Immunology, Corporate Member of Freie Universität Berlin and Humboldt-Universität zu Berlin, Charité – Universitätsmedizin Berlin, Augustenburger Platz 1, 13353 Berlin, Germany

**Keywords:** Post-COVID, Long-COVID, Glucocorticoids, Treatment, Methylprednisolone, Placebo-controlled trial protocol, Pathophysiology

## Abstract

**Introduction:**

Post-COVID-19 Syndrome (PCS) includes neurological manifestations, especially fatigue and cognitive deficits. Immune dysregulation, autoimmunity, endothelial dysfunction, viral persistence, and viral reactivation are discussed as potential pathophysiological mechanisms. The post-corona-virus immune treatment (PoCoVIT) trial is a phase 2a randomized, controlled, double-blind trial designed to evaluate the effect of methylprednisolone versus placebo on cognitive impairment in PCS. This trial is designed based on the hypothesised autoimmunological pathogenesis and positive aberrations, employing a series of off-label applications.

**Methods:**

Recruitment criteria include a diagnosis of PCS, a minimum age of 18 years and self-reported cognitive deficits at screening. A total of 418 participants will be randomly assigned to either verum or placebo intervention in the first phase of the trial. The trial will consist of a first trial phase intervention with methylprednisolone versus placebo for six weeks, followed by a six-week treatment interruption period. Subsequently, an open second phase will offer methylprednisolone to all participants for six weeks. Outpatient follow-up visits will take place two weeks after each trial medication cessation. The third and final follow-up, at week 52, will be conducted through a telephone interview. The primary outcome measures an intra-patient change of 15 or more points in the memory satisfaction subscale of the Multifactorial Memory Questionnaire (MMQ) from baseline to follow-up 1 (week 8). Key secondary outcomes include long-term intra-patient changes in memory satisfaction from baseline to follow-up 2 (week 20), changes in other MMQ subscales (follow-up 1 and 2), and changes in neuropsychological and cognitive scores, along with assessments through questionnaires focusing on quality of life, fatigue, and mood over the same periods. Exploratory outcomes involve molecular biomarkers variations in serum and cerebrospinal fluid, as well as structural and functional brain magnetic resonance imaging (MRI) parameters changes related to cognition.

**Perspective:**

This trial aims to contribute novel evidence for treating patients with PCS, with a primary focus on those manifesting cognitive deficits. By doing so, it may enhance comprehension of the underlying pathophysiological mechanisms, thereby facilitating biomarker research to advance our understanding and treatment of patients with PCS.

## Introduction

In late 2019, a new strain of the coronavirus-family, severe acute respiratory syndrome coronavirus 2 (SARS-CoV-2), emerged. It spread rapidly around the world and precipitated the Coronavirus Disease 2019 (COVID-19) pandemic, thereby significantly impacting global public health [13]. Consequently, many patients continue to experience protracted symptoms, commonly referred to as ‘long-COVID’ or post-COVID-19 syndrome (PCS), a condition that poses a substantial burden to society. PCS exhibits an overall estimated prevalence ranged from 7.5 to 41% in non-hospitalized adults and 37.6% in hospitalized adults [18]. The manifestation of PCS is more common among younger and female patients [3, 6]. PCS is defined by the WHO as a persistent condition, lasting at least three months after infection with broad spectrum of symptoms which may affect all organ systems and impairing daily activity [22]. Cardinal neurological symptoms include fatigue and cognitive deficits [5], which impose a significant impact on quality of life [22]. Patients report a range of cognitive deficits such as concentration difficulties, word-finding difficulties, memory lapses, attention problems and “brain fog”. Systematic neuropsychological assessments reveal deficits in processing speed, executive function, phonemic fluency, category fluency, and memory encoding [2].

Pathophysiological mechanisms underlying the development of PCS and persistent neurological manifestations following SARS-CoV-2 infection revolve around ongoing viral persistence and direct viral invasion, reactivation of latent herpesviridae, endothelial/microcirculatory dysfunction, neuroinflammation, and autoimmunological processes [9, 15, 16, 21]. These potential pathomechanisms involve the overactivation of the immune system, characterized by hyperinflammation and cytokine release. A notable contributor is the post-viral induction of autoimmunity, a well-established mechanism observed in other diseases [16, 21], alongside molecular mimicry, where anti-pathogen antibodies cross-react with host proteins [19].

Based on previous findings, we propose that SARS-CoV-2 triggers an immune response resulting in a dysregulated autoimmune system involving both cellular and humoral components. Preliminary studies of our research group have shown an association of neuronal antibodies present in both serum and cerebrospinal fluid (CSF) and pathological montreal cognitive assessment (MoCA) results in patients with cognitive deficits [9, 16, 21]. Reactive autoantibodies in the central nervous system (CNS), detected in both serum and CSF during SARS-CoV-2 infection, have been consistently observed in a significant proportion of patients [7, 10, 12, 17]. While the role of biomarkers as indicators for CNS damage or involvement in pathophysiological processes remains challenging, they represent potential targets for immunosuppressive treatment. The rationale behind an autoimmune genesis hypothesis suggests that methylprednisolone could be a viable treatment approach. This well-established anti-inflammatory glucocorticoid has proven efficacy in treating various diseases suspected to have an autoimmune aetiology, characterized by inflammatory changes or immune overactivity. Examples include multiple sclerosis, chronic inflammatory demyelinating polyradiculoneuropathy, and steroid-responsive autoantibody related encephalitis [14]. While some of these diseases can be cured completely with corticosteroids, others require repeated pulse therapy. There are several controlled trials that employ corticosteroids, particularly methylprednisolone, at similar doses for autoimmune neurological diseases [8, 24]. The use of glucocorticoids has been explored in acute SARS-CoV-2 infections associated with an excessive inflammatory response [1, 11]. Methylprednisolone holds promise in disrupting various immunological triggers, presenting a well-known, generally well-tolerated, cost-effective, and widely available therapeutic option [14]. Our data indicates that off-label administration of methylprednisolone resulted in subjective improvements and enhanced performance on neuropsychological tests in a high proportion of PCS patients with CSF autoantibodies (data in preparation). This underscores the potential efficacy of the chosen treatment approach within this specific patient cohort. To date, no randomized, placebo-controlled trials have examined the effects of methylprednisolone in patients with PCS primarily characterized by cognitive deficits.

## Methods

### Objective of the trial

The post-corona-virus immune treatment (PoCoVIT) trial aims to examine the effect of methylprednisolone versus placebo in patients with cognitive deficits in PCS syndrome in a randomized controlled double-blind trial. Additionally, the trial includes exploratory research to identify potential biomarkers, given the frequent occurrence of CNS reactive autoantibodies in the serum and CSF of patients with PCS, particularly those with neurological symptoms such as cognitive deficits. The PoCoVIT trial may enhance our understanding of treatment strategies, thereby improving patient care. Simultaneously, it contributes to biomarker research advancing our understanding of the disease’s pathomechanisms. The objective is not only to demonstrate improvements in clinically relevant and qualitative patient-oriented measures from baseline to week 8 but also to establish correlations between these measures and objective antibody-related mechanistic indicators derived from baseline CSF, neuroimaging, and biomarkers. The second treatment phase, wherein all patients receive methylprednisolone will provide further insights into whether repetitive corticosteroid pulse therapy proves beneficial in symptom control among patients with PCS.

### Trial description and design

**Fig. 1:**
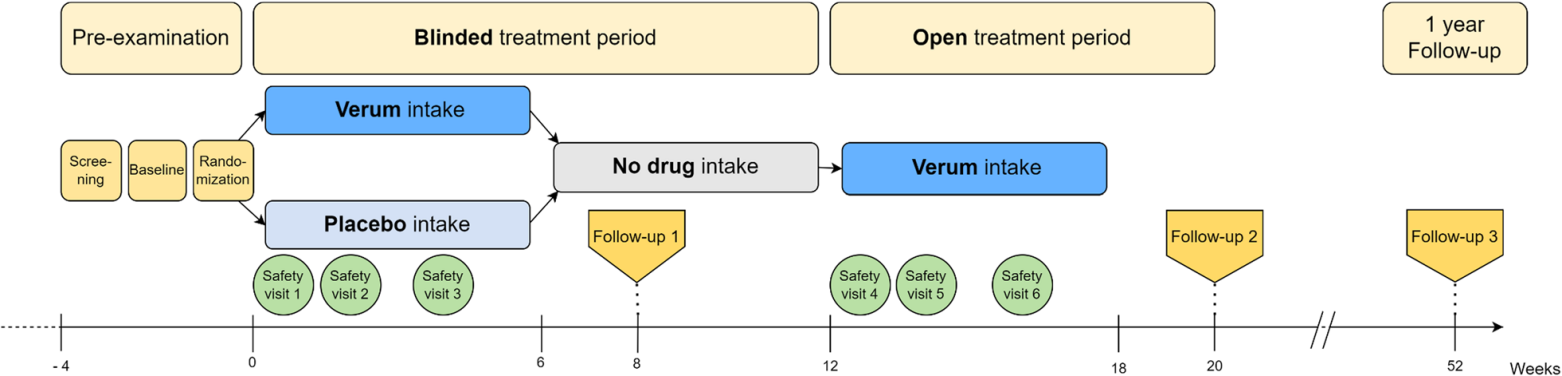
Flowchart of the post-corona-virus immune treatment (PoCoVIT) trial

The PoCoVIT trial is a prospective, single centre trial (OECD category 2) employing a randomized, double blind, placebo-controlled, 2-arm parallel-group design. Patients with predominantly cognitive deficits are enrolled. Figure 1 provides an overview of the main trial procedures. Methylprednisolone serves as the active treatment (verum), and our hypothesis centres on achieving clinically significant improvement following its administration. The primary endpoint is the intra-patient change in Multifactorial Memory Questionnaire (MMQ) subdomain memory satisfaction by ≥ 15 points from baseline to week 8 [25].

Eleven trial visits, detailed in Table 1, including screening, are planned for each patient. The total trial duration for each participant is one year, including two treatment phases, each consisting of 4 weeks of treatment followed by 2 weeks of tapering.

**Table 1:** Visit schedule with assessments

Period/Visit name	Screening	Baseline 1	Baseline 2 &Randomization	Treatment period: Placebo vs Verum^1^			Follow-Up	Treatment period: Verum^2^			Follow-Up	
	S (V1)	B1	B2 (V2)	SV (V3)	SV (V4)	SV (V5)	FU1 (V6)	SV (V7)	SV (V8)	SV (V9)	FU2 (V10)	FU3 (V11)
Trial days	− 28 to 0		− 14 to 0	2	14	28	56	86	98	112	140	365
Time windows	± 3 days											± 30 days
Outpatient visit	•		•	•	•	•	•	•	•	•	•	
Informed consent	•											
Inclusion and exclusion criteria	•											
Randomization			•									
Discharge from trial											•	
*History*												
Demographic data			•									•
Medical history			•									
*Diagnostic assessments*												
Structural cMRI		•										
Diagnostic blood sample^3^			•				•				•	
Lumbar puncture^4^			•									
*Safety assessments*												
Neurological and general physical examination			•				•				•	
Pregnancy test in female patients			•		•		•		•		•	
Blood sample A^5^				•	•	•		•	•	•		
Blood sample B^6^			•				•				•	
Clinical and sonographic exclusion of thrombosis					•				•			
AE questioning			•	•	•	•	•	•	•	•	•	
Concomitant medication			•	•	•	•	•	•	•	•	•	•
*Outcome assessments*												
Patient reported symptoms			•				•				•	•
Multifactorial Memory Questionnaire			•				•				•	•
Short form 36 Health survey			•				•				•	•
Fatigue severity scale			•				•				•	•
Chalder fatigue scale			•				•				•	•
Beck depression inventory-II			•				•				•	
Montreal cognitive assessment			•				•				•	
PROMIS 29			•				•				•	
PROMIS Cognitive Function Short form 4a			•				•				•	
Verbal learning and memory test			•				•				•	
Brief Visuospatial memory test—revised			•				•				•	
WAIS-IV			•				•				•	
Test battery for attention performance			•				•				•	
Trail-making-test A and B			•				•				•	
Regensburger Wortflüssigkeits-test			•				•				•	
Symbol digit modalities test			•				•					
Wortschatztest			•									
LPS			•									
Assessment of self-experienced exposure to IMP						•						
Enrolment in diagnostic and biomarker platform study in a subgroup			•				•					

AE, Adverse Event; cMRI, cranial magnetic resonance imaging; LPS, Performance testing system (Leistungsprüfungssystem: Subtest 3: Logical Thinking); PROMIS, Patient reported outcomes measurement information system; WAIS-IV, Digit span (forward/backward) from the Wechsler Adult Intelligence Scale-Fourth Edition

^1^Treatment period: Placebo vs Verum begin at day 1 ± 3

^2^Treatment period: Verum begin at day 85 ± 3

^3^Blood sample: autoantibodies, complement system and inflammatory markers

^4^Lumbar puncture: autoantibody testing and inflammatory markers

^5^Blood sample A: Glucose, Na, K, GFR, creatinine

^6^Blood sample B: Differential blood count, glucose, Na, K, HbA1c, GFR, creatinine, TSH, AST, ALT

Patients receive trial information and undergo screening before enrolment. At baseline 1 (B1), participants are scanned using cerebral MRI. At baseline 2 and prior to randomization (B2/R), patients complete a neurological and neuropsychological assessment. Blood and optional CSF samples are collected and assessed potential occurrence of autoantibodies. Selected patients, with or without autoantibodies, may be enrolled, following informed consent, in the biomarker and diagnostic platform receiving additional blood analyses and imaging. The biomarker and diagnostic platforms within our centre serve as databases for the identification of diagnostic and prognostic markers associated with PCS, using both imaging data and blood/CSF samples. After randomization, treatment phase 1 unfolds over six weeks, during which patients are randomly assigned to either verum (methylprednisolone) or placebo treatment. Follow-up 1 occurs two weeks post the cessation of trial medication of treatment phase 1. After a four-week treatment break, the second treatment phase begins with all patients receiving methylprednisolone for an additional six weeks. Follow-up 2 occurs two weeks post the cessation of treatment phase 2. Follow-up 3, scheduled 52 weeks after randomization, comprises a telephone interview and patient-completed questionnaires.

During both treatment phases, each participant undergoes safety and monitoring assessments involving blood tests and ultrasound examination for exclusion of deep vein thrombosis of the lower extremity. Detailed documentation of demographic data, medical history, and clinical assessments is conducted during the trial visits (for details refer to Table 1).

### Eligibility criteria

Patients with PCS and cognitive deficits will be recruited from the specialised outpatient clinic of the Department of Neurology at Charité. Inclusion and exclusion criteria for the trial population are comprehensively outlined in Table 2. Informed consent from all participants is mandatory and will be documented after the screening visit, ensuring at least a 24-h period before baseline 1.

**Table 2:** Inclusion and exclusion criteria for trial population

*Inclusion criteria*
1. History of confirmed (PCR or serology) SARS-CoV-2 infection according to WHO criteria
2. Ongoing symptoms of PCS for ≥ 3 months
3. Self-reported predominant cognitive deficits at screening
4. Male or female adult who is 18 years or older at the time of informed consent
5. Subject is willing, understanding, and able to provide informed consent
6. Signed informed consent prior to initiation of any trial related measure
7. For female subject or diverse subjects:
(a) Confirmed post-menopausal state, defined as amenorrhea for at least 12 months, or
(b) If being of childbearing potential:
1. Negative highly sensitive urine or serum pregnancy test before inclusion, and
2. Practicing a highly effective birth control method (failure rate of less than 1%):
(a) combined (oestrogen and progestogen containing) hormonal
(b) contraception associated with inhibition of ovulation (oral/intravaginal/ transdermal), or
(c) progestogen-only hormonal contraception associated with inhibition of ovulation (oral/injectable/implantable), or
(d) Intrauterine device, or
(e) Intrauterine hormone-releasing system, or
(f) Bilateral tubal occlusion, or
(g) Vasectomised partner, or
(h) Heterosexual abstinence
*Exclusion criteria*
1. Any ongoing central nervous system disease
2. Any major psychiatric disease within the last 10 years
3. Previous medical history of gastric ulcer, osteoporosis and/or previous vertebral fractures, rheumatological disease or metabolic disease including diabetes mellitus
4. Ongoing immunosuppressive therapy
5. Patient is pregnant or breastfeeding at screening
6. MMQ memory satisfaction subdomain > 50 points at Screening
7. Current malignant disease (including space-occupying brain tumours)
8. Body weight < 45 kg
9. Severe lactose intolerance
10. Participation in another clinical interventional trial within the last 3 months or five half-lives of the other trial’s IMP, if longer than 6 months previous to informed consent
11. Patient is institutionalized by order of court or public authority
12. Patient who might be dependent on the sponsor, the investigator or the trial site
13. Place of living does not allow the subject to attend the planned study visits
14. Other conditions that are likely to affect the safety of the study treatment (e.g., severely impaired immune status)
*Additional exclusion criteria for MRI assessments*
1. Pacemaker
2. Metal implants and other objects that are not proven MRI safe (e.g., drug pumps, heart valves, shards, joint prosthesis, hearing aids, stents)

IMP, Investigational medicinal product; MMQ, Multifactorial Memory Questionnaire; MRI, magnetic resonance imaging; PCR, Polymerase chain reaction; PCS, Post-COVID-19 syndrome; SARS-CoV-2, Severe acute respiratory syndrome coronavirus 2; WHO, World Health Organization

### Arms and intervention

Participants will be stratified based on age (> 50 years or ≤ 50 years), sex and cognitive screening assessment results, as determined by the MoCA at baseline 2 (> 26/30 or ≤ 26/30 points). Randomization will occur in a 1:1 ratio, assigning participants to either the verum or placebo group. The total intervention duration per participant is 20 weeks. The initial six weeks constitute a double-blind intervention with a starting dose of body weight-adjusted methylprednisolone or matching placebo. In the verum group, participants receive oral methylprednisolone at a dose of approximately 1 mg/kg body weight, taken once daily for four weeks followed by a two-week tapering phase. Conversely, in the control group participants receive a matching placebo orally once daily for six weeks, adhering to a comparable titration schedule to maintain blinding. Following the initial six-week blinded intervention, a drug-free period of six weeks ensues. This is followed by a second, unblinded treatment phase lasting the same duration with an equivalent tapering scheme. During this phase, all participants receive methylprednisolone Three follow-up visits (week 8, week 20, and week 52) are scheduled in addition to the six safety visits.

### Outcome measures

The primary outcome measure is the intra-patient change in the MMQ sub-domain memory satisfaction, demonstrating an increase of ≥ 15 points from baseline to week 8 [25]. Key secondary and safety outcome measures include: (a) Intra-patient change in MMQ sub-domain memory satisfaction from baseline to week 20 and week 52, along with changes from week 8 to week 20, and from week 20 to week 52 (b). Mean difference in MMQ sub-domain memory performance and memory strategy from baseline to week 8, (c) Intra-patient change in neuropsychological and cognitive scores and quality of life measures from baseline to week 8 and week 20, and from week 8 to week 20 (d). Exploratory biomarker-related outcomes encompass the occurrence and change in molecular biomarkers in serum and CSF as well as structural or functional alterations in imaging of cortical and subcortical regions and fibre tracts implicated in neurocognitive processes.

The assessment of investigational medicinal product side effects is conducted through adverse event, serious adverse event, and suspected or unexpected serious adverse reaction reports. Detailed objective measures and outcomes are shown in Table 3.

**Table 3:** Objectives and Outcomes

Objective	Outcome
*Primary*	
Improvement in memory satisfaction from baseline to week 8 when comparing methylprednisolone with placebo in PCS patients	Intra-patient change in MMQ subdomain memory satisfaction by ≥ 15 points from baseline to week 8
*Secondary*	
Long-term improvement in memory satisfaction from baseline to week 20 and to week 52	Intra-patient change in MMQ subdomain “memory satisfaction” from baseline to week 20, from week 8 to week 20 and from week 20 to week 52
Improvement in memory ability, and memory strategy	Mean difference in MMQ subdomain “memory ability” from baseline to week 8 and to week 20 and from week 8 to week 20
Mean difference in MMQ subdomain “memory strategy” to week 8 and to week 20 and from week 8 to week 20	
Improvement in neurocognitive symptoms quantified using neuropsychological and cognitive scores	Intra-patient change in neuro-psychological and cognitive scores (MoCA, neuropsychological test battery, SDMT) from baseline to week 8 and to week 20 and from week 8 to week 20
Improvement in quality of life	Intra-patient change in quantified PROMIS questionnaire and SF-36 from baseline to week 8 and to week 20 and to week 52 and from week 8 to week 20
Improvement in fatigue and mood	Intra-patient change in fatigue scores (FSS, CFQ) and mood (BDI) from baseline to week 8 and to week 20 and to week 52 and from week 8 to week 20
*Key safety*	
Infections meeting SAE criteria	Occurrence of IMP side and adverse effects, assessed with AE, SAE and SUSAR reports
Psychiatric complications (depression, euphoria, severe sleep disorders)	
Endocrinologic disorders meeting SAE criteria	
*Exploratory*	
Biomarker-related: Autoimmune activity, immune status, inflammatory biomarkers, complement system	Occurrence and changes in molecular biomarkers
Biomarker-related: Structural changes in the brain and in brain functions	Structural and functional alterations in MRI imaging in cortical and subcortical regions and fibre tracts with relevance to neurocognitive processes

AE, adverse event; BDI, beck depression inventory II; CFQ, chandler fatigue scale; FSS, fatigue severity scale; IMP, investigational medicinal product; MMQ, Multifactorial Memory Questionnaire; MoCA, Montreal Cognitive Assessment; MRI, magnetic resonance imaging; PROMIS, patient reported outcomes measurement information system; SAE, serious adverse event; SDMT, symbol digit modalities test; SF-36, Short form 36 health survey; SUSAR, Suspected unexpected serious adverse reaction

### Sample size calculation and statistical analysis

For the PoCoVIT trial, we aim to detect an improvement in the MMQ sub-domain memory satisfaction. We consider a change of additional 15 points a clinically relevant improvement. Based on our previous data, we anticipate this improvement in 15% of the verum group and 5% of the placebo group. To achieve 90% power at a significance level of α = 0.05, we require 188 patients in each group. This calculation uses a two-sample test for proportions (z-test with arcsine transformation) in the stats package of the R statistical computing environment (version 4.0.2). Assuming a dropout rate of 10%, our recruitment target is 209 patients per group, ensuring that 188 participants remain after dropout. We, therefore, plan to screen 700 patients to enrol the necessary 418, stratified by sex, age, and MoCA score.

Since a single primary outcome is selected, no multiplicity adjustment is required. Analyses of secondary outcomes will be exploratory. For the primary outcome, a binary logistic regression model will be used, focusing on improvement in outcome variables and treatment group as main effects, adjusting for stratification variables. Odds ratios will be reported with 95% confidence intervals, and an alpha of 0.05 has been set for significance. Complete case analysis will be conducted. Where missing values exceed 5%, sensitivity analysis will be performed using multiple imputation chain equations [4].

Secondary outcomes will be initially analysed descriptively and modelled using appropriate techniques such as ANCOVA and logistic models, depending on the outcome distributions. The treatment variable will be included as the main factor adjusting for stratification variables similar to the primary analysis model. Treatment effects will be calculated with two-sided 95% confidence intervals, and outcomes will be analysed in complete cases analyses. Safety, subgroup and interim analyses will be conducted. Statistical analyses will be performed using SAS release 9.2 or higher (SAS Institute Inc., Cary, North Carolina, USA) and R (www.r-project.org) version 4.1.2 or higher.

### Contacts

The PoCoVIT trial is conducted within the Nationale Klinische Studiengruppe (NKSG), a clinical trial and translational research platform for the development of treatment in PCS and ME/CFS, funded by the German Ministry of Education and Research (BMBF) [20].

### Perspective

This trial is based on the hypothesis that an immune system overactivation triggers sustained hyperinflammation and autoantibody production in PCS. Autoantibodies, including those against neurotransmitter receptors, have been identified in several cohorts of patients with PCS [9, 10]. The identification and specification of relative frequencies are still limited due to the broad spectrum of antibodies. Notably, autoantibodies against G protein-coupled receptors (GPCRs), particularly ß2- and a1-adrenoreceptors, angiotensin II-, muscarinic M2-, MAS-, nociception–, and ETA-receptors, have been found in sera from PCS patients with neurological and/or cardiac symptoms [26]. These autoantibodies correlated with symptom severity (e.g., fatigue, vasomotor and cognitive symptoms) in patients with post-acute sequelae of COVID-19 with chronic fatigue syndrome (PACS-CFS) [23]. Our findings indicate that autoantibodies targeting brain epitopes are common in patients with PCS and strongly associated with pathological cognitive screening test, especially when detected in CSF [9, 10]. However, due to the recent nature of this research, the epitopes and nature of these autoantibodies have not yet been fully characterized.

Evidence regarding the effect of immunosuppressive therapeutics is urgently needed. To date there are no proven therapeutic options to treat patients with PCS, which imposes a significant burden on individuals, society, and the economy. Methylprednisolone, chosen as the active treatment in this trial, is a cost-effective pharmaceutical with demonstrated clinical effectiveness in various immune-mediated diseases. Adverse effects are well described, and safety visits are scheduled accordingly in the PoCoVIT trial. An additional major aim of PoCoVIT is to enhance our understanding of the pathophysiological mechanisms underlying the disease paving the way for potential therapeutic approaches. In PCS, the imperative for adequately powered randomized placebo-controlled trials is evident, seeking to replace controversial experimental therapeutic approaches with evidence-based decision making in the future.

## Data Availability

The original contributions presented in the trial are included in the article/supplementary material, further inquiries can be directed to the corresponding author.

## Abbreviations

B1: Baseline 1
B2/R: Baseline 2 and randomization
CNS: Central nervous system
CSF: Cerebrospinal fluid
COVID-19: Coronavirus disease 2019
GPCRs: G protein-coupled receptors
MRI: Magnetic resonance imaging
MoCA: Montreal cognitive assessment
MMQ: Multifactorial Memory Questionnaire
PoCoVIT: Post-corona-virus immune treatment
PCS: Post-COVID 19 Syndrome
SARS-CoV-2: Severe acute respiratory syndrome coronavirus 2

## References

[1] Angus DC, Derde L, Al-Beidh F, Annane D, Arabi Y, Beane A, van Bentum-Puijk W, Berry L, Bhimani Z, Bonten M, Bradbury C, Brunkhorst F, Buxton M, Buzgau A, Cheng AC, de Jong M, Detry M, Estcourt L, Fitzgerald M, Summers C (2020). Effect of hydrocortisone on mortality and organ support in patients with severe COVID-19: The REMAP-CAP COVID-19 corticosteroid domain randomized clinical trial. JAMA.

[2] Becker JH, Lin JJ, Doernberg M, Stone K, Navis A, Festa JR, Wisnivesky JP (2021). Assessment of cognitive function in patients after COVID-19 infection. JAMA Network Open.

[3] Boesl F, Audebert H, Endres M, Pruss H, Franke C (2021). A neurological outpatient clinic for patients with post-COVID-19 syndrome - A report on the clinical presentations of the first 100 patients. Frontiers in Neurology.

[4] Buuren S, Groothuis-Oudshoorn C (2011). MICE: multivariate imputation by chained equations in R. Journal of Statistical Software.

[5] Ceban F, Ling S, Lui LMW, Lee Y, Gill H, Teopiz KM, Rodrigues NB, Subramaniapillai M, Di Vincenzo JD, Cao B, Lin K, Mansur RB, Ho RC, Rosenblat JD, Miskowiak KW, Vinberg M, Maletic V, McIntyre RS (2022). Fatigue and cognitive impairment in post-COVID-19 syndrome: A systematic review and meta-analysis. Brain, Behavior, and Immunity.

[6] Chen C, Haupert SR, Zimmermann L, Shi X, Fritsche LG, Mukherjee B (2022). Global prevalence of post-coronavirus disease 2019 (COVID-19) condition or long COVID: A meta-analysis and systematic review. Journal of Infectious Diseases.

[7] Delamarre L, Gollion C, Grouteau G, Rousset D, Jimena G, Roustan J, Gaussiat F, Aldige E, Gaffard C, Duplantier J, Martin C, Fourcade O, Bost C, Fortenfant F, Delobel P, Martin-Blondel G, Pariente J, Bonneville F, Geeraerts T, Neuro ICURG (2020). COVID-19-associated acute necrotising encephalopathy successfully treated with steroids and polyvalent immunoglobulin with unusual IgG targeting the cerebral fibre network. Journal of Neurology, Neurosurgery and Psychiatry.

[8] Flanagan EP, McKeon A, Lennon VA, Boeve BF, Trenerry MR, Tan KM, Drubach DA, Josephs KA, Britton JW, Mandrekar JN, Lowe V, Parisi JE, Pittock SJ (2010). Autoimmune dementia: Clinical course and predictors of immunotherapy response. Mayo Clinic Proceedings.

[9] Franke C, Boesl F, Goereci Y, Gerhard A, Schweitzer F, Schroeder M, Foverskov-Rasmussen H, Heine J, Quitschau A, Kandil FI, Schild AK, Finke C, Audebert HJ, Endres M, Warnke C, Pruss H (2023). Association of cerebrospinal fluid brain-binding autoantibodies with cognitive impairment in post-COVID-19 syndrome. Brain, Behavior, and Immunity.

[10] Franke C, Ferse C, Kreye J, Reincke SM, Sanchez-Sendin E, Rocco A, Steinbrenner M, Angermair S, Treskatsch S, Zickler D, Eckardt KU, Dersch R, Hosp J, Audebert HJ, Endres M, Ploner JC, Pruss H (2021). High frequency of cerebrospinal fluid autoantibodies in COVID-19 patients with neurological symptoms. Brain, Behavior, and Immunity.

[11] Group RC, Horby P, Lim WS, Emberson JR, Mafham M, Bell JL, Linsell L, Staplin N, Brightling C, Ustianowski A, Elmahi E, Prudon B, Green C, Felton T, Chadwick D, Rege K, Fegan C, Chappell LC, Faust SN, Landray MJ (2021). Dexamethasone in hospitalized patients with COVID-19. The New England Journal of Medicine.

[12] Guilmot A, Maldonado Slootjes S, Sellimi A, Bronchain M, Hanseeuw B, Belkhir L, Yombi JC, De Greef J, Pothen L, Yildiz H, Duprez T, Fillee C, Anantharajah A, Capes A, Hantson P, Jacquerye P, Raymackers JM, London F, El Sankari S, van Pesch V (2021). Immune-mediated neurological syndromes in SARS-CoV-2-infected patients. Journal of Neurology.

[13] Hu B, Guo H, Zhou P, Shi ZL (2021). Characteristics of SARS-CoV-2 and COVID-19. Nature Reviews Microbiology.

[14] McDaneld LM, Fields JD, Bourdette DN, Bhardwaj A (2010). Immunomodulatory therapies in neurologic critical care. Neurocritical Care.

[15] Meinhardt J, Radke J, Dittmayer C, Franz J, Thomas C, Mothes R, Laue M, Schneider J, Brunink S, Greuel S, Lehmann M, Hassan O, Aschman T, Schumann E, Chua RL, Conrad C, Eils R, Stenzel W, Windgassen M, Heppner FL (2021). Olfactory transmucosal SARS-CoV-2 invasion as a port of central nervous system entry in individuals with COVID-19. Nature Neuroscience.

[16] Monje M, Iwasaki A (2022). The neurobiology of long COVID. Neuron.

[17] Mulder J, Lindqvist I, Rasmusson AJ, Husen E, Ronnelid J, Kumlien E, Rostami E, Virhammar J, Cunningham JL (2021). Indirect immunofluorescence for detecting anti-neuronal autoimmunity in CSF after COVID-19—Possibilities and pitfalls. Brain, Behavior, and Immunity.

[18] Nittas V, Gao M, West EA, Ballouz T, Menges D, Wulf Hanson S, Puhan MA (2022). Long COVID through a public health lens: An umbrella review. Public Health Reviews.

[19] Nunez-Castilla J, Stebliankin V, Baral P, Balbin CA, Sobhan M, Cickovski T, Mondal AM, Narasimhan G, Chapagain P, Mathee K, Siltberg-Liberles J (2022). Potential autoimmunity resulting from molecular mimicry between SARS-CoV-2 spike and human proteins. Viruses.

[20] Scheibenbogen C, Bellmann-Strobl JT, Heindrich C, Wittke K, Stein E, Franke C, Pruss H, Pressler H, Machule ML, Audebert H, Finke C, Zimmermann HG, Sawitzki B, Meisel C, Toelle M, Krueger A, Aschenbrenner AC, Schultze JL, Beyer MD, Burock S (2023). Fighting post-COVID and ME/CFS—Development of curative therapies. Frontiers in Medicine.

[21] Song E, Bartley CM, Chow RD, Ngo TT, Jiang R, Zamecnik CR, Dandekar R, Loudermilk RP, Dai Y, Liu F, Sunshine S, Liu J, Wu W, Hawes IA, Alvarenga BD, Huynh T, McAlpine L, Rahman NT, Geng B, Farhadian SF (2021). Divergent and self-reactive immune responses in the CNS of COVID-19 patients with neurological symptoms. Cell Rep Med.

[22] Soriano JB, Murthy S, Marshall JC, Relan P, Diaz JV (2022). A clinical case definition of post-COVID-19 condition by a Delphi consensus. The Lancet Infectious Diseases.

[23] Sotzny F, Filgueiras IS, Kedor C, Freitag H, Wittke K, Bauer S, Sepulveda N, Mathias da Fonseca DL, Baiocchi GC, Marques AHC, Kim M, Lange T, Placa DR, Luebber F, Paulus FM, De Vito R, Jurisica I, Schulze-Forster K, Paul F, Scheibenbogen C (2022). Dysregulated autoantibodies targeting vaso- and immunoregulatory receptors in post COVID syndrome correlate with symptom severity. Frontiers in Immunology.

[24] Strupp M, Zingler VC, Arbusow V, Niklas D, Maag KP, Dieterich M, Bense S, Theil D, Jahn K, Brandt T (2004). Methylprednisolone, valacyclovir, or the combination for vestibular neuritis. New England Journal of Medicine.

[25] Troyer AK, Rich JB (2002). Psychometric properties of a new metamemory questionnaire for older adults. Journals of Gerontology. Series B, Psychological Sciences and Social Sciences.

[26] Wallukat G, Hohberger B, Wenzel K, Furst J, Schulze-Rothe S, Wallukat A, Honicke AS, Muller J (2021). Functional autoantibodies against G-protein coupled receptors in patients with persistent Long-COVID-19 symptoms. Journal of Translational Autoimmunity.

